# Analysis of association of potentially functional genetic variants within genes encoding miR-34b/c, miR-378 and miR-143/145 with prostate cancer in Serbian population

**DOI:** 10.17179/excli2019-1257

**Published:** 2019-07-16

**Authors:** Nevena Kotarac, Zorana Dobrijevic, Suzana Matijasevic, Dusanka Savic-Pavicevic, Goran Brajuskovic

**Affiliations:** 1 Centre for Human Molecular Genetics, Faculty of Biology, University of Belgrade, Belgrade, Serbia

**Keywords:** prostate cancer, miRNA, rs4938723, rs1076064, rs4705342, association study

## Abstract

MiRNA-associated genetic variants occurring in regulatory regions can affect the efficiency of transcription and potentially modify pri-miRNA or pre-miRNA processing. Since miRNA-based mechanisms are shown to be involved in the pathogenesis of prostate cancer (PCa), the aim of the present study was to evaluate the effect of rs4938723, rs1076064 and rs4705343 occurring in regulatory regions of miR-34b/c, miR-143/145 and miR-378, respectively, on PCa risk and progression in Serbian population. We examined a total of 1060 subjects, of which 350 were patients with PCa, 354 were patients with benign prostatic hyperplasia (BPH), while 356 healthy volunteers were included in the control group. Genotyping of rs4938723, rs1076064 and rs4705343 was performed by using Taqman^®^ SNP Genotyping Assays. Allele C of rs4705342 was found to increase the risk of PCa (*P*=0.031 for codominant model, *P*=0.0088 for recessive model). Rs1076064 minor allele G was found to associate with serum PSA score, as well as with PCa T category and disease aggressiveness. For rs4938723 minor allele C was shown to be associated with the lower PCa T category (P_dom_=0.0046; OR=0.36, 95 % CI 0.17-0.76) in T2 vs. T1 comparison. Rs4705342 was identified as PCa susceptibility variant in Serbian population, while for rs1076064 and rs4938723 association with PCa progression parameters was found.

## Introduction

Prostate cancer (PCa) is the most common cancer among men in Europe. Based on the most recent data from WHO and Eurostat databases, predicted mortality from PCa in Europe for 2018 was 77.000 deaths, which is about 7 % higher than observed in 2012 (Szeliski et al., 2018[34]). In Serbia, PCa is the third most commonly diagnosed cancer in men, according to the data obtained by the National Institute of Public Health. At the same time, in the period between 1991 and 2015, the upward trend of mortality was determined for major cancer types, with the largest increase in cancer mortality rates demonstrated for PCa (Institute of Public Health of Serbia “Dr Milan Jovanović Batut”, 2014[19]). Striking statistics have led to focusing attention of both medical practitioners and scientists on the improvements in diagnostic procedures and patient monitoring. Despite the recent advances in diagnostic procedures and therapeutic approaches, PCa still suffers from overdiagnosis and overtreatment and novel molecular markers are urgently needed for better management and risk stratification of patients (Loeb et al., 2014[24]). 

Among the genetic variants discovered as PCa susceptibility loci, most originated from genome-wide association studies while the others located within important tumor-suppressor genes or oncogenes involved in molecular basis of PCa were discovered through candidate-gene based approach (Nikolić et al., 2016[26]). Recently, microRNA-related genes have emerged as novel candidates, due to accumulating evidence supporting the involvement of regulatory RNA species in PCa pathogenesis (Kanwal et al., 2017[21]). MicroRNAs (miRNAs) are a class of small, evolutionarily conserved non-coding RNAs about ~23 nt in length that post-transcriptionally regulate gene expression. MicroRNAs regulate almost all basic cellular functions, from cell differentiation to cell cycle-arrest and apoptosis. At posttranscriptional level miRNAs negatively regulate protein expression of oncogenes or tumor-supressive genes and could participate in carcinogenesis as either tumor-supressor or oncogenic miRNAs (Felekkis et al., 2010[11]). Therefore, genetic variants occuring in different constituents of miRNA network, from miRNA genes themselves to miRNA binding sites and miRNA-processing machinery, could potentially be implicated in molecular pathogenesis of PCa. MiRNA-associated genetic variants occurring in regulatory regions can affect the efficiency of transcription and, depending on their location relative to mature microRNA, potentially modify pri-miRNA or pre-miRNA processing. Also, variants changing the sequence of mature microRNA, as well as variants located in miRNA-binding site within mRNA, could influence the binding affinity, or even create/destroy binding sites. Furthermore, genetic variants affecting processing machinery potentially disrupt proper maturation (Ryan, 2017[30]). Based on these functional implications, case-control studies, as well as case-only studies among PCa patients, could lead to possible identification of miRNA-related variants associated with PCa susceptibility and/or progression risk. Any new molecular marker identified through this approach and validated in larger independent cohorts has potential to be implemented in diagnostic or prognostic algorithms aiming to facilitate identification of high-risk patients, as well as to improve the management of therapeutic process (Rupaimoole and Slack, 2017[29]). 

In our previous reports, we have investigated the association of common genetic variants within mature microRNAs with PCa risk and progression (Nikolic et al., 2014[28], 2015[25]). Furthermore, we have analyzed the potential effect of rs875919 located in the terminal loop of miR-27a precursor (Nikolic et al., 2015[25]). These results encouraged us to further examine the impact of genetic variants with potential effect on microRNA function on the risk of PCa development and tumor aggressiveness. Among the novel candidate genetic variants for the present study is rs4938723, which lies within the potential regulatory region of pri-miR-34b/c and is previously reported to be related to higher risk of PCa (Hashemi et al., 2017[14]). This potentially functional variant is associated with the risk of cervical, hepatocellular cancer (HCC), childhood acute lymphoblastic leukemia (AML), gastric cancer and esophageal squamose cell carcinoma (ESCC) (Xu et al., 2018[36]). Similarly, genetic variant rs1076064 which could affect the transcription and/or processing of pri-miR-378 is related to several types of cancer including breast cancer, renal cell cancer (RCC) and AML (Ding et al., 2018[9]). However, to date, there are no reports regarding the impact of this variant on PCa risk. Since miR-143 and miR-145 are among the most commonly downregulated miRNAs in neoplasms such as gastric, urinary bladder, colorectal cancer, as well as PCa, genetic variants potentially affecting the expression of their precursor have been investigated in multiple malignant diseases. Among a number of SNPs located within the potential regulatory region of miR-143/145 cluster, the effect on transcriptional actions was revealed for rs4705343 and rs4705342 (Wei et al., 2016[35], Sima et al., 2017[32]). Also, a previous report suggests the association of genetic variant rs4705342 with PCa (Chu et al., 2016[7]). Furthermore, a functional assay has shown the effect of rs4705342 on transcriptional regulation (Chu et al., 2016[7]). 

The association of genetic variants rs4705342 and rs4938723 with PCa was previously assessed in single studies conducted in populations of China and Iran, respectively. Therefore, the aim of the present study is to evaluate the effect of these genetic variants on PCa risk and progression in a population of European origin. Also, as far as we are aware, the present case-control study is the first one aiming to assess the possible association of a common cancer-related genetic variant rs1076064 with PCa. 

## Material and Methods

The study used peripheral blood samples obtained from the collections of DNA samples from the Center for Human Molecular Genetics. The collection consisted of patients treated in the period between 2008 and 2013 at Clinical Centre “Dr Dragiša Mišović Dedinje”, Belgrade, Serbia and Clinical Centre “Zvezdara”, Belgrade, Serbia (Brankovic et al., 2013[3][4]; Nikolic et al., 2014[27][28], 2015[25]). Research was conducted with the approval of ethics committees of these medical institutions. The study was done in accordance with the Helsinki Declaration. 

This case-control study examined a total of 1060 subjects: 350 peripheral blood samples from patients with sporadic PCa, 354 samples from patients with benign prostatic hyperplasia (BPH) as well as 356 buccal swab or peripheral blood samples from healthy control individuals. Controls were recruited after passing standard annual physical examination. PCa and BPH were diagnosed by means of standard clinical procedures which combined digital rectal examination (DRE), transrectal ultrasonography (TRUS), abdominal and pelvic ultrasound, serum prostate-specific antigen (PSA) level, bone scintigraphy and radiography, and finally prostate biopsy.

Patients with PCa were selected into groups based on the values of standard prognostic parameters: Prostate specific antigene (PSA) at diagnosis (PSA<10 ng/ml; 10 ng/ml≤ PSA< 20 ng/ml; PSA>20 ng/ml), Gleason score (GS) (GS<7; GS=7; GS>7) and T category, according to TNM staging (T1; T2; T3/T4). Also, three groups of patients with different risk of localized cancer progression were formed, according to criteria recommended by European Association of Urology (EAU) (Heidenreich et al., 2014[17]). Groups were defined as low-risk (PSA<10 ng/ml, GS<7, and T category T1-T2a), intermediate-risk (PSA 10-20 ng/ml or GS=7 or T category T2b-T2c), and high-risk (PSA>20 ng/ml or GS>7 or T category T3/T4). Since patients with metastases were included in the study, the criteria were modified to include this subset into high risk group.

QIAamp® DNA Mini Kit (*QIAGEN*, Hilden, Germany) and Blood DNA Isolation Mini Kit, (*Norgen Biotek Corp.*, Canada) were used for isolation of genomic DNA from peripheral blood and buccal swab samples, following the manufacturers' protocol. Genotyping of rs4938723, rs1076064 and rs4705342 was performed using Taqman^®^ SNP Genotyping Assays (Applied Biosystems, Foster City, California, USA). Statistical analysis of SNPs associations was performed by using SNPStats software (Sole et al., 2006[33]). Hardy-Weinberg equilibrium (HWE) was assessed using exact test implemented in SNPStats software. Allelic and genotypic associations were evaluated by unconditional logistic regression method with adjustment for age. Separate comparisons were done for five different genetic models: allelic (log-additive), codominant, dominant, recessive, and overdominant. Odds ratio (OR) and its 95 % confidence intervals (95 % CI) were used as risk estimates. The best fitting models were determined by using Akaike information criterion (AIC). All tests were two-sided, and P value less than 0.05 was considered as statistically significant.

## Results

Clinical and pathological characteristics of PCa patients are presented in Table 1(Tab. 1). According to available data, the most frequently determined initial serum PSA scores among PCa patients were higher than 20 ng/ml. Also, over a half of PCa patients had GS=6 (54 %), as well as TNM T category T2 of primary cancer (55.1 %), while distant metastases were found in 16 % of PCa patients at diagnosis. 

Allele and genotype frequencies of rs4938723, rs1076064 and rs4705342 in PCa patients, BPH patients and healthy controls are summarized in Table 2(Tab. 2). Genotype distributions among controls were consistent with Hardy-Weinberg equilibrium (*P*=0.91, *P*=0.26 and *P*=0.6, for rs4938723, rs1076064 and rs4705342, respectively).

In the comparison of genotype distributions and allele frequencies of tested genetic variants among PCa patients and healthy controls no statistically significant differences were found. Conversely, when comparing genotype distributions among PCa and BPH patients, allele C of rs4705342 was found to increase the risk of PCa (*P*=0.031 for codominant model, *P*=0.0088 for recessive model). The exact OR could not be calculated, since CC genotype was not found in BPH patients (Table 2(Tab. 2)).

By comparing genotype distributions among PCa patients with initial serum PSA scores 10 ng/ml≤ PSA< 20 ng/ml and PSA<10 ng/ml, rs1076064 minor allele G was found to confer the increased risk of higher PSA score under dominant genetic model (P_dom_=0.032; OR_dom_=1.89, 95%CI 1.05-3.41) (Table 3(Tab. 3)). The analysis of association between other analyzed genetic variants and the serum PSA scores did not show statistical significance for any genetic model tested (results not shown). 

When genotype frequencies among PCa patients with different GSs were compared, statistical significance was not reached for any genetic variant tested. Nevertheless, the comparison of genotype distributions among PCa patients with GS =7 and GS<7 showed the statistical trend for the association between rs1076064 minor allele G and higher GS (P=0.082, for dominant genetic model). Statistical trend of association was also found in the comparison of rs1076064 genotype frequencies among PCa patients with GS>7 and GS =7, but with the opposite direction of the effect of minor allele G (P_dom_=0.09 and P_log-additive_=0.081) (Table 4(Tab. 4)).

By comparing genotype frequencies among subgroups of PCa patients with TNM T categories T1 and T2, rs4938723 minor allele C was found to be associated with the lower category (P_dom_=0.0046; OR=0.36, 95 % CI 0.17-0.76) (Table 5(Tab. 5)). Beside the best-fitting dominant genetic model, statistical significance was obtained for association between rs4938723 and PCa T category for codominant, overdominant and log-additive models (*P*_codom_=0.015, *P*_overdom_=0.011, *P*_log-additive_=0.037). Also, statistical trend of significance was reached for the association between rs4938723 and the disease stage in the comparisons of genotype frequencies between PCa patients with T3/4 and T1 T categories, as well as between patients with T3/4 and T2. As for other genetic variants tested, minor allele G of rs1076064 was found to associate with the lower primary PCa stage, when comparing genotype distributions among patients stratified into groups with T3/4 and T2 T categories (*P*_rec_=0.0083, OR=0.34, 95 % CI 0.14-0.81) (Table 5(Tab. 5)). Also, the genetic variants tested were not shown to be associated with the presence of distant metastases among PCa patients (results not shown). 

When patients were stratified according to the risk of cancer progression, rs1076064 minor allele G was found to be associated with higher PCa aggressiveness (Table 6(Tab. 6)). Statistical significance was reached for multiple genetic models of association in the comparison of genotype frequencies among PCa patients with high-risk and low-risk disease (*P*_codom_=0.017, *P*_dom_=0.0068, *P*_overdom_=0.011, *P*_log-additive_=0.033), as well as among patients with medium-risk and low-risk disease (*P*_codom_=0.0073, *P*_dom_=0.0024, *P*_overdom_=0.0073, *P*_log-additive_=0.014). The lowest AIK score in both comparisons was determined for dominant genetic model.

## Discussion

Regulatory activities of non-coding RNA molecules have been extensively studied in the last decade, including their involvement in molecular pathogenesis of human malignancies. Therefore, evidence obtained through these studies has accumulated to support the role of dysregulation of microRNA-based mechanisms in various phases of malignant transformation and metastasis (Ryan et al., 2010[31]). Among the most extensively studied miRNAs in the context of human carcinogenesis are the members of miR-34 family, microRNAs transcribed from miR-143/145 cluster, as well as miR-378. Data indicating the importance of these microRNAs in the onset and progression of malignancies were obtained in the analyses of expression of both microRNAs and their targets in neoplastic and paired normal cells. Also, numerous studies aimed to functionally analyze potentially oncogenic or tumor-suppressive microRNAs through monitoring the effects of their experimental silencing and/or the augmentation of their expression on malignant phenotypes (Di Leva et al., 2014[8]). Since genetic variants were found to affect miRNA transcription efficiencies and/or miRNA processing, target selection and target silencing efficiency, microRNA genes have been recognized as candidates for genetic association studies on human cancer, including PCa (Hayes et al., 2014[16]). Since tumor-suppressive roles for miR-34b/c, miR-143/145 and miR-378 have been previously demonstrated in PCa (Zhou et al., 2015[40]; Chen et al., 2016[6]; Fang et al., 2017[10]), we speculated that genetic variants with the potential effect on their expression and/or processing could play significant roles in PCa development and/or progression.

One of the most studied genetic variants with the potential effect on the expression of miR-34 family members is rs4938723. This variant locates upstream of the region encoding the shared common pri-microRNA for mature miR-34b and miR-34c and is predicted to influence their biogenesis by affecting the transcription rate of precursor microRNAs. According to *in silico* analysis, rs4938723 resides within a CpG island of *hsa-miR-34b/c* gene and potentially influences the binding of GATA transcription factors to the regulatory region (Xu et al., 2011[37]). The regulatory importance of this genetic variant was further augmented by the luciferase reporter gene assay results from human embryonic kidney 293 cells (293T cell line), showing the increased luciferase activity of the vector with the rs4938723 T allele relative to the vector with C allele (Zhang et al., 2014[39]). 

To date, over 25 case-control studies investigated this genetic variant and its association with cancer risk and progression, yielding contrasting results. The vast majority of these studies were conducted in Asian populations, and the most recent meta-analysis suggests the association of rs4938723 with hepatocellular (HCC) and colorectal cancer, esophageal squamous cell carcinoma, as well as acute lymphoblastic leukemia (Hashemi et al., 2019[15]). According to the scientific literature database search, only one study conducted in Iranian population analyzed the potential association between genetic variant rs4938723 and PCa (Hashemi et al., 2017[14]). Therefore, we aimed to conduct a case-control study on the association between rs4938723 and risk of PCa onset and/or progression, which would provide the first data on this issue in a European population. 

Hashemi et al. found that this genetic variant associates with the risk of PCa under several genetic models tested (Hashemi et al., 2017[14]). In contrast to these results, we found no evidence to support the association of rs4938723 with PCa susceptibility. The observed discrepancy may be due to differences in ethnic backgrounds, which are possibly reflected by the allele frequencies substantially differing between Serbian and Iranian populations. Another explanation for the inconsistent findings may be due to previous study's relatively small sample size, with about two times smaller number of PCa patients and controls compared to ours (Hashemi et al., 2017[14]). On the other hand, Hashemi et al. found no statistically significant association of rs4938723 with clinical characteristics of their PCa patients, which is concordant with our results showing no evidence of correlation between rs4938723 genotypes and initial serum PSA score and GS. Nevertheless, in the present study, allele C was found to associate with the lower risk of localized PCa progression to the higher T category. Even though the number of PCa patients stratified to single TNM-stage based categories was relatively small in the present study, the difference between our results and the previously obtained in Iranian population could be attributed to the discordant staging system, since Hashemi et al. used pathological instead of clinical tumor staging (Hashemi et al., 2017[14]). 

Genetic variant rs1076064 locates 222 bp upstream from the region encoding the stem-loop precursor of miR-378. Due to its position regarding the predicted transcription start site (TSS) of miR-378, this genetic variant was suggested to potentially alter the expression of mature miR-378 through affecting the transcriptional rate and/or its processing of pri-miR-378. In an attempt to test such relation, a study conducted by An et al. (2014[1]) has shown that G allele of rs1076064 exerts higher promoter activity than allele A in the luciferase reporter gene assay in multiple hepatocellular carcinoma (HCC) cell lines. Furthermore, the position of this genetic variant corresponds to the c-Myc-binding site predicted by Feng et al., who also identified c-Myc as a regulator of the transcriptional unit containing both miR-378 and its host gene *PPARGC1B* (Feng et al., 2011[12]). Recently, another study provided evidence to support the regulatory features of the sequence located upstream of the region encoding the stem-loop precursor of miR-378, containing the rs1076064 locus, by identifying a potential intronic promoter of miR-378 within this region (Jiang et al., 2014[20]). 

The study by An et al. demonstrated the decreased risk of HCC, as well as better prognosis, associated with rs1076064 allele G (An et al., 2014[1]). The same research group previously found the association of rs1076064 with Non-Small Cell Lung Cancer patient survival (Hu et al., 2011[18]). Also, this genetic variant was found to be associated with the age of diagnosis among pancreatic cancer patients (Chen et al., 2016[5]). To our knowledge, the association of this genetic variant with PCa risk and progression was not previously tested. Still, its potential functional significance related to miR-378 expression regulation justifies the inclusion of rs1076064 in the present study, since miR-378 was found to be deregulated in PCa. Namely, Avgeris et al. found that miR-378 level was reduced in PCa compared to benign gland lesions (Avgeris et al., 2014[2]). The same study also demonstrated that the downregulation of miR-378 correlated with high GS and higher serum PSA levels and the ability of miR-378 to predict survival outcomes of actively treated patients (Avgeris et al., 2014[2]). 

Our result did not support the association of rs1076064 with PCa risk. On the other hand, minor allele G was found to associate with higher PSA score, as determined by comparing genotype frequencies between PCa patients with intermediate and low initial serum PSA scores. When comparing genotype distributions among patients stratified into groups with T3/4 and T2 TNM categories, minor allele G associated with lower T category. The opposing direction of association with different prognostic parameters could be explained by the relatively small number of patients within some groups after stratification. Beside these statistically significant associations, statistical trend was reached in comparison of rs1076064 genotype distributions among patients with different GSs. When patients were stratified according to the risk of cancer progression, rs1076064 minor allele G was found to be associated with higher PCa aggressiveness in Serbian population. Nevertheless, these results need to be taken with caution due to the small number of PCa patients included in the group with low risk for disease progression. Since our study provide unique data on the relation between rs1076064 and PCa, our results could not be compared with any previously obtained in other populations. 

Another genetic variant included in the present study is rs4705342, located upstream of the region encoding miR-143. Similarly as for rs1076064, this genetic variant was proposed to alter the expression of miR-143 by affecting the transcriptional rate and/or processing of pri-miR-143/145, which also serves as a precursor of miR-145, another important cancer-related microRNA. Through luciferase reporter gene assays, the effect of allelic variants of rs1076064 on miR-143 expression was shown in both HCC and PCa cell lines (Chu et al., 2016[7]; Yin et al., 2018[38]). Also, the presence of C allele was found to increase the affinity of NF-kB for its binding site (Yin et al., 2018[38]). 

Liang et al. demonstrated that rs4705342 is associated with the risk of cervical squamous cell carcinoma in Chinese women (Liang et al., 2015[23]). Also, this genetic variant was found to associate with the breast cancer recurrence among patients from Iran (Ghanbarpanah et al., 2018[13]). In contrast to these results, rs4705342 was not confirmed as colorectal cancer susceptibility variant in Chinese Han population (Li et al., 2013[22]). As for the PCa study conducted by Chu et al. found that this potentially functional genetic variant contributes PCa susceptibility in Chinese population. In this previous study, the protective effect was attributed to allele C (Chu et al., 2016[7]). Conversely, our results support the association of rs4705342 minor allele C with the increased risk of developing PCa, as shown in the comparison of genotype distribution between PCa and BPH patients. The lack of statistical significance in the comparison of PCa patients and healthy controls can be explained by the far better clinical characterization of BPH patients, making them supercontrols in this type of research. Among the reasons for the observed discordances in the results of the present and the previous study could be the difference in ethnic backgrounds of study populations, as well as unmatched subject recruitment procedure. Namely, in our study, the control group consisted of healthy male subjects, while for the study by Chu et al. control subjects were defined as cancer-free and were recruited while seeking health care. Furthermore, our results did not replicate the association of rs4705342 with standard prognostic parameters of PCa found by Chu et al. (2016[7]). Still, in this previous study, subgrouping of patients based on clinicopathological characteristics differed from ours. Also statistical testing was not matching, since genotype distributions in PCa patients subgroups were compared to controls and not to each other (Chu et al., 2016[7]).

Even though over 1000 participants were included in this study, its main limitation is a relatively small sample size. Also, stratification of PCa patients according to standard prognostic parameters of PCa progression yielded several small subgroups. Therefore, caution should be taken when interpreting the results obtained in comparisons involving these small subgroups of patients. The observed discrepancies in results of previous studies and our present study could be explained by differences in ethnic origins, as well as in the recruitment of participants and genotyping procedures. In order to elucidate the association of the tested genetic variants with PCa and to verify our findings, further case-control studies with larger sample sizes and in different ethnicities are needed.

Even though this study has severe limitations, our results identified rs4705342 as PCa susceptibility variant in Serbian population. At the same time, for genetic variants rs1076064 and rs4938723 association with PCa progression parameters was found. 

## Acknowledgements

We would like to thank the following persons for their contribution to the research: Dr Vinka Vukotic, MD PhD and Dr Natasa Filipovic, MD from the Department of Urology, Clinical Centre “dr Dragiša Mišović”, Belgrade, Serbia, Dr Snezana Cerovic, MD PhD from the Institute of Pathology, Military Medical Academy, Belgrade, Serbia and last but not least to Dr Sasa Tomovic, MD from Clinical Department of Surgery, Clinical Center “Zvezdara,” Belgrade, Serbia. The research was supported by the Ministry of Education, Science and Technological Development of the Republic of Serbia (Project no. 173016). 

## Conflict of interest

The authors declare that they have no conflict of interest.

## Figures and Tables

**Table 1:**
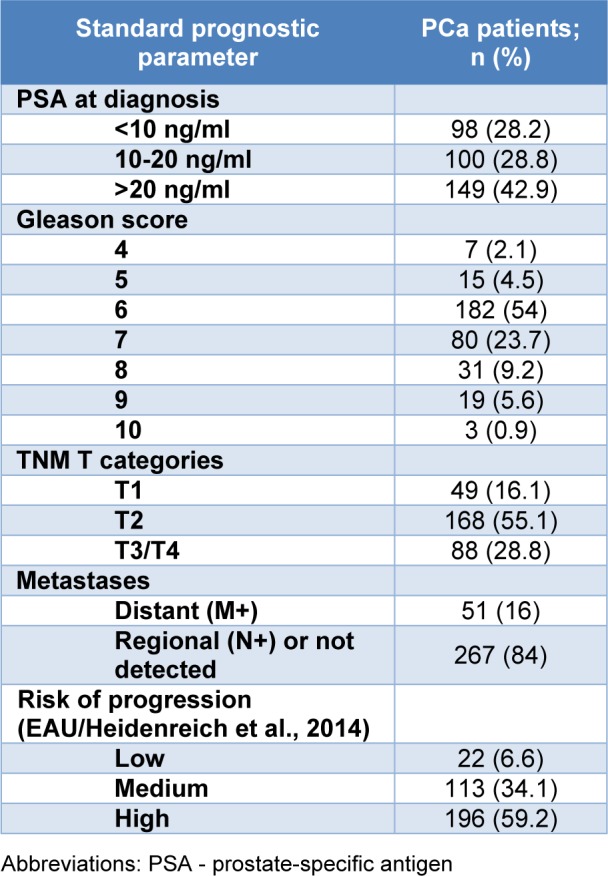
Classification of patients with PCa based on the values of standard prognostic parameters of disease progression, presence of distant metastases and the risk of cancer progression.

**Table 2:**
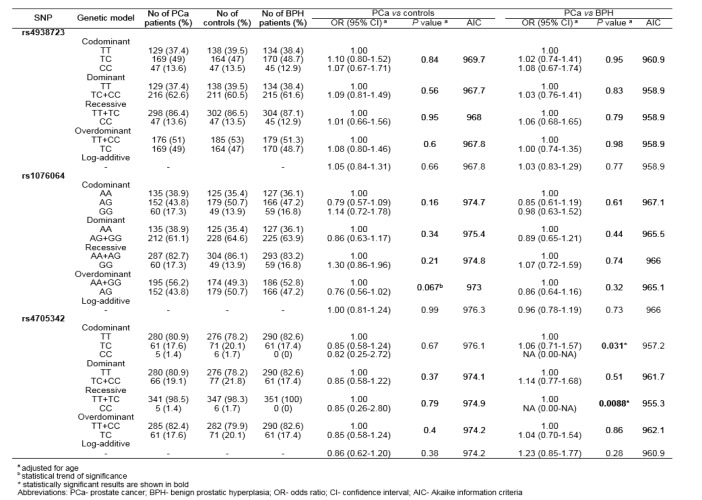
Association of genetic variants within genes encoding miR-34b/c, miR-378 and miR-143/145 with PCa risk

**Table 3:**
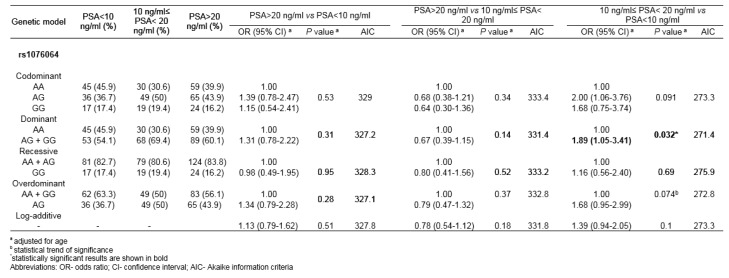
Association of rs1076064 with initial serum PSA scores

**Table 4:**
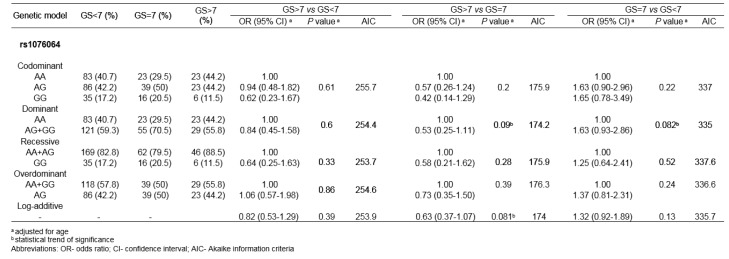
Association of rs1076064 with Gleason score

**Table 5:**
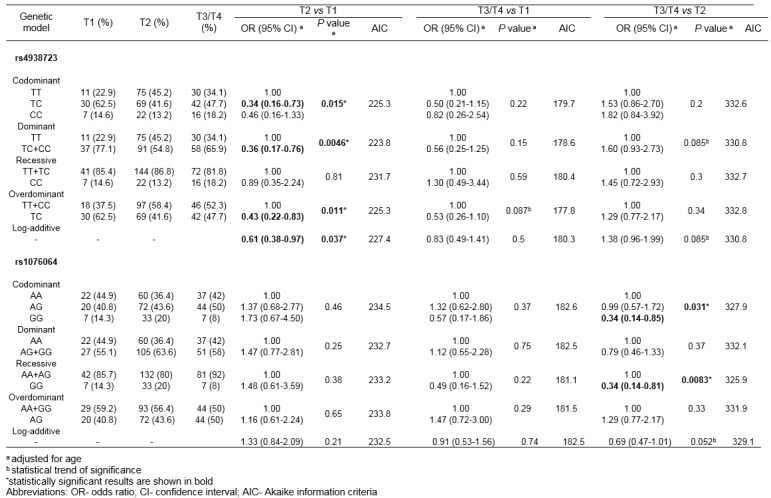
Association of rs1076064 and rs4938723 with the TNM categories of localized PCa

**Table 6:**
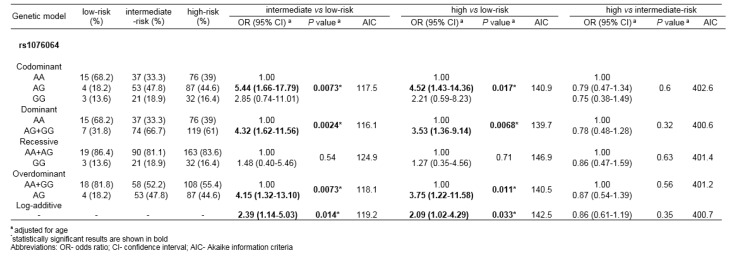
Association of rs1076064 with the risk of cancer progression
